# TRANSITION TO A MORE EVEN DISTRIBUTION OF PROTEIN INTAKE IS ASSOCIATED WITH ENHANCED FAT LOSS IN OBESE OLDER ADULTS

**DOI:** 10.1093/geroni/igz038.3097

**Published:** 2019-11-08

**Authors:** Samaneh Farsijani, Jane A Cauley, Adam J Santanasto, Nancy W Glynn, Robert M Boudreau, Anne B Newman

**Affiliations:** 1 University of Pittsburgh, Pittsburgh, Pennsylvania, United States; 2 University of Pittsburgh, Department of Epidemiology, Pittsburgh, Pennsylvania, United States

## Abstract

Background: Optimization of intentional weight loss in obese older adults, through preferential fat mass reduction, is challenging, as the concomitant lean mass loss may exacerbate sarcopenia. Here, we assessed whether changes in within-day protein intake distribution are related to improvements in body composition in overweight/obese older adults during a hypocaloric and exercise intervention. Methods: Thirty-six community-dwelling, overweight-to-obese (BMI 28.0-39.9 kg/m2), sedentary older adults (aged 70.6±6.1 years) were randomized into either physical activity plus successful aging health education (PA+SA; n=15) or physical activity plus weight loss (PA+WL; n=21) programs. Body composition (by CT and DXA) and dietary intake (by three-day food records) were determined at baseline, 6-month, and 12-month follow-up visits. Within-day protein distribution was calculated as the coefficient of variation of protein ingested at breakfast [5:00–10:59], lunch [11:00–16:59] and dinner [17:00–1:00]. Secondary analysis was performed to determine associations between changes in protein intake distribution and body composition. Results: In both groups, baseline protein intake was skewed towards dinner. The pattern of protein intake changed towards a more even within-day distribution in PA+WL, but it remained unchanged in PA+SA. Transition towards a more even pattern of protein intake was independently associated with a greater decline in BMI (P<0.05) and abdominal subcutaneous fat (P<0.05) in PA+WL. However, changes in protein CV were not associated with weight loss in PA+SA. Conclusion: Our results show that mealtime distribution of protein intake throughout the day was associated with improved weight and fat loss under hypocaloric diet combined with physical activity.

